# The prefrontal cortex of the bottlenose dolphin (*Tursiops truncatus* Montagu, 1821): a tractography study and comparison with the human

**DOI:** 10.1007/s00429-023-02699-8

**Published:** 2023-09-03

**Authors:** Tommaso Gerussi, Jean-Marie Graïc, Antonella Peruffo, Mehdi Behroozi, Lara Schlaffke, Stefan Huggenberger, Onur Güntürkün, Bruno Cozzi

**Affiliations:** 1 Department of Comparative Biomedicine and Food Science (BCA), University of Padua, Legnaro, Italy; 2 Department of Biopsychology, Institute of Cognitive Neuroscience, Faculty of Psychology, Ruhr-University Bochum, 44801 Bochum, Germany; 3 Department of Neurology, BG-University Hospital Bergmannsheil, Ruhr-University Bochum, Bürkle-de-La-Camp-Platz 1, 44789 Bochum, Germany; 4 Institute of Anatomy and Clinical Morphology, Witten/Herdecke University, Alfred-Herrhausen-Straße 50, 58448 Witten, Germany; 5 Research Center One Health Ruhr, Research Alliance Ruhr, Ruhr-University Bochum, Bochum, Germany

**Keywords:** Cetacean, Brain evolution, Dolphin, PFC, CSD, DWI

## Abstract

Cetaceans are well known for their remarkable cognitive abilities including self-recognition, sound imitation and decision making. In other mammals, the prefrontal cortex (PFC) takes a key role in such cognitive feats. In cetaceans, however, a PFC could up to now not be discerned based on its usual topography. Classical in vivo methods like tract tracing are legally not possible to perform in Cetacea, leaving diffusion-weighted imaging (DWI) as the most viable alternative. This is the first investigation focussed on the identification of the cetacean PFC homologue. In our study, we applied the constrained spherical deconvolution (CSD) algorithm on 3 T DWI scans of three formalin-fixed brains of bottlenose dolphins (*Tursiops truncatus*) and compared the obtained results to human brains, using the same methodology. We first identified fibres related to the medio-dorsal thalamic nuclei (MD) and then seeded the obtained putative PFC in the dolphin as well as the known PFC in humans. Our results outlined the dolphin PFC in areas not previously studied, in the cranio-lateral, ectolateral and opercular gyri, and furthermore demonstrated a similar connectivity pattern between the human and dolphin PFC. The antero-lateral rotation of the PFC, like in other areas, might be the result of the telescoping process which occurred in these animals during evolution.

## Introduction

**Fig. 1:**
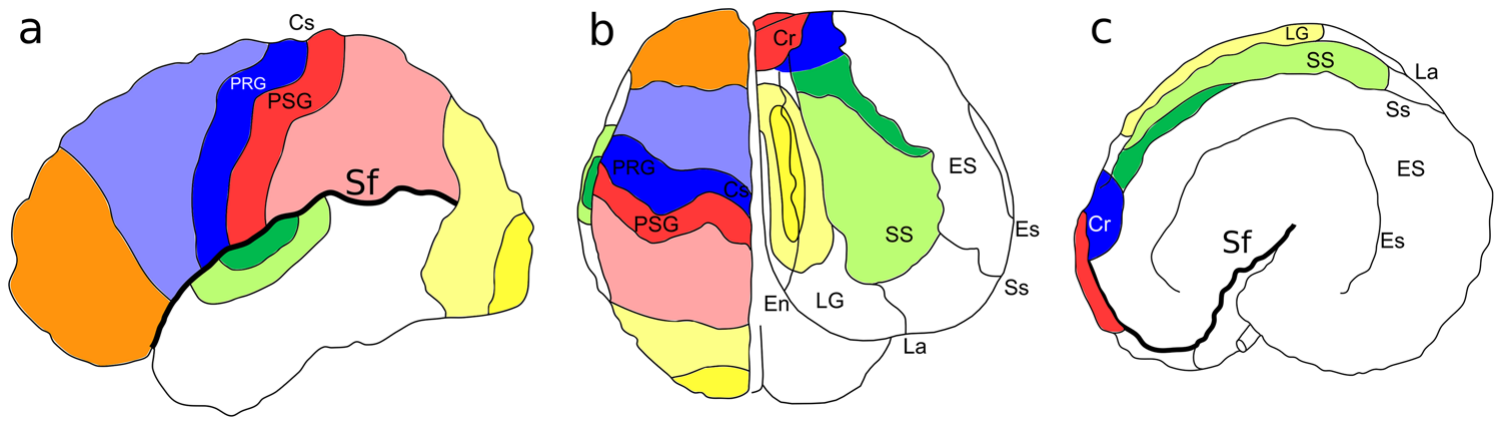
Neocortical brain map of **a** human in left view, **b** human (left hemisphere) and dolphin (right hemisphere) in dorsal view and **c** dolphin in left view. Dark blue, Motor cortex; light blue, premotor cortex; Red, somatosensory cortex; light red, associative somatosensory cortex; yellow, V1; light yellow, associative visual cortex; dark green, A1; light green, associative auditory cortex; orange, PFC. *Cr* cruciate sulcus, *Cs* central sulcus, *En* entolateral sulcus, *ES* ectosylvian gyrus, *Es* ectosylvian sulcus, *La* lateral sulcus, *LG* lateral gyrus, *PRG* precentral gyrus, *PSG* postcentral gyrus, *Sf* Sylvian fissure, *SS* suprasylvian gyrus, *Ss* suprasylvian sulcus. Neocortical maps adapted from Martin (2021; human) and Cozzi et al. (2017, dolphin)

The bottlenose dolphin *Tursiops truncatus* (Montagu, 1821) is a member of the *Delphinidae* family often kept in captivity, and consequently frequently studied. Individuals of this species may perform a large variety of complex cognitive tasks including sound imitation, understanding of human syntax, conceptual decision taking, understanding numerosity, and self-recognition (Kilian et al. 2003; Kuczaj et al. 2009; Herman 2012; Yaman et al. 2012; Güntürkün 2014; Loth et al. 2022). Bottlenose dolphins demonstrated a capacity for complex planning and to devise composite hunting strategies (Tyack 2009; Herman 2012; Loth et al. 2022). In terrestrial mammals, these executive functions, elaborate behavioural actions, and the required working memory, are regulated by the prefrontal cortex (PFC; Fuster 2015), a neocortical area deemed responsible for higher brain functions in humans (Tranel et al. 2003; Butler and Hodos 2005; Kandel et al. 2021). The PFC is the “cortex of the anterior pole of the brain” and defined as the major receiver of thalamic inputs from the medio-dorsal nucleus (MDN) (Petrides and Pandya 2012; Fuster 2015). The MDN indeed projects also to the cingulate, insular premotor and parietal cortices (May and Forutan 2012). The identification of the PFC, as for other areas, is, therefore, based on composite anatomical landmarks and functional experiments that result in well-accepted cortical maps of the human brain compared to the dolphin brain (Fig. 1a, b). The topographical concept of PFC has been applied to the brain of lab rodents, domestic carnivores, sheep, rabbit, and primates, and validated functionally mostly through invasive studies (Rose and Woolsey 1948; Dinopoulos et al. 1985; Fuster 2015).

The brain of the bottlenose dolphin weighs approx. 1600 g and has roughly 3700 cm^2^ of cortical surface, which are higher absolute values compared to the human brain (1300 g and 2400 cm^2^, respectively; Ridgway and Brondson 1984; Hofman 1985; Cozzi et al. 2017). The current neocortical maps for this species (See Chapter 6 in Cozzi et al. 2017; Chapter 5 in Huggenberger et al. 2019) derive almost entirely from early direct, intracortical evoked potential studies (Lende and Akdikmen 1968; Ladygina and Supin 1970, 1974; Lende and Welker 1972; Sokolov et al. 1972; for review, see Bullock and Gurevich 1979; Supin et al. 2001; Cozzi et al. 2017) and retrograde tracing (Garey and Revishchin 1990). These pioneering studies allowed the tentative identification and preliminary topography of key functional areas, including motor (MC), somatosensory (SSC), primary and secondary auditory (A1, A2), and primary and secondary visual (V1, V2) cortices (Fig. 1b, c). No further invasive studies were published afterwards, due to growing ethical concerns and public awareness of animal rights. A large part of the dolphin cerebral cortex remains, therefore, unexplored, and several functions are still not mapped topographically.

The absence of an experimentally based topographical identification of the associative areas of the bottlenose dolphins (and other cetacean species) represents a potential critical point that limits neuroanatomical comparisons and potentially hampers even behavioural studies (for discussion, see Chapter 10 in Cozzi et al. 2017). To date, the PFC has not been delineated in dolphins, and the topography of the rostral portion of the cortex in dolphins does not clearly match that of terrestrial mammals, including artiodactyls. What has been stated so far is that the antero-ventral (“frontal” or “orbital”) part of the dolphin brain contains very large pyramidal cells, attributable to a putative motor cortex (see Kojima 1951; Hof et al. 2005). However, the variety and complexity of behaviours briefly described above suggest the existence of an area at least partially functionally homologous to the human PFC. To further support this hypothesis, here we emphasise that the thalamic nuclei (including the MDN) that in primates and rodents are usually related to associative cortical targets, are markedly developed in the dolphin (Kruger 1959, 1966). A recent publication on the brains of the spotted and common dolphins (Berns et al. 2015) described a thalamo-temporal pathway, based on the use of Diffusion Tensor Imaging (DTI). The striatal projections from the basal ganglia to the “frontal” lobes were investigated as well and a similar connectivity pattern to primates was found. Nevertheless, these findings were presented as a validation of the technique compared to the other data analysed and no further assumption about a putative PFC in cetaceans was present (Berns et al. 2015).

Invasive tracing studies in live dolphins are now ethically unacceptable, and similar considerations may prevent MR studies of healthy dolphins anaesthetised only for research purposes. Furthermore, many research and clinical MRI bores and coils may be unfit for the large head of the species. Consequently, DTI and other diffusion-weighted imaging (DWI) techniques on whole-fixed brains represent one of the few ethical and practical approaches to study connectivity patterns, but their applications to dolphins have been rare.

DTI was the first mathematical algorithmic method to encode DWI in a 3D tensor model with 6 degrees of freedom and perform tractography. Since the tensor model only represents the most dominant diffusion orientation, crossing fibres are not considered (Basser 1995). To overcome this known problem, a new algorithm, the Constrained Spherical Deconvolution (CSD), was developed. CSD is based on the acquisition of High Angular Resolution Diffusion Imaging (HARDI) and its model includes the possibility of multiple dominant diffusion orientations as in crossing fibres and, therefore, allows a better understanding of the organisation of fibre tracts (Tournier et al. 2007; Calamuneri et al. 2018).

CSD and other DWI methods are mathematical models that provide an interpretation of biological barriers within the brain. These methods are susceptible to artefacts, and the resulting data must be carefully interpreted. Tractography algorithms cannot distinguish between inputs and outputs, or rather, do not add a sense to the direction of the fibres, and as such, the words “terminated” (or similar) should be interpreted bearing in mind this caveat. Despite this limitation, DWI methods are currently the only non-invasive techniques available for studying structural brain connectivity (Schilling et al. 2020).

In the present study, we investigated whether the bottlenose dolphins possess an area with the topographical and connection characteristics of the human PFC, as detected by CSD. To this effect, we acquired post-mortem DW images on three adult bottlenose dolphin brains and performed tractography using CSD to investigate the fibre pathways involving the MDN. The results were compared with parallel investigations on the human brain.

## Materials and methods

### Origin of the specimens

Information on the bottlenose dolphin (*n* = 3) and human (*n* = 5) brains used in the present study are reported in Table 1.

**Table 1:** Origin of specimens

Species	ID	Sex	Age	Origin	Cause of death	Fixation interval	Imaging interval
*T. truncatus*	# 4	F	Adult	Marine theme park	Drowning	< 12 h	21 yr
	# 9	F		Marine theme park	Septicaemia	< 12 h	20 yr
	# 457	M		Wild	NA	< 12 h	2 yr
*H. sapiens*	MGH 1007	M	Adult	Not applicable	NA	NA	NA
	MGH 1010	F					
	MGH 1016	M					
	MGH 1019	F					
	MGH 1031	M					

The age class was known in the captive animals and estimated in the wild due to its total length (Jefferson et al. 2015)

Dolphin brains were extracted during routine necropsy performed at the Department of Comparative Biomedicine and Food Science (BCA) of the University of Padova (Italy) on specimens stranded on the Veneto coast. The brains were consequently fixed in phosphate buffered paraformaldehyde (4%) and stored in the *Mediterranean marine mammal tissue bank* (MMMTB, http://www.marinemammals.eu), located in BCA. The MMMTB is a CITES recognised (IT020) research centre, sponsored by and collaborating with the Italian Ministry of the Environment and the University of Padova. MMMTB collects and stores samples from wild or captive marine mammals whose samples or whole carcasses are delivered to BCA for post-mortem diagnostics.

Alive human brains were obtained from the Human Connectom Project (HCP) database (https://ida.loni.usc.edu/login.jsp). HCP is supported by the National Institute of Dental and Craniofacial Research (NIDCR), the National Institute of Mental Health (NIMH) and the National Institute of Neurological Disorders and Stroke (NINDS). The HCP is the result of efforts of co-investigators from the University of Southern California, Martinos Center for Biomedical Imaging at Massachusetts General Hospital (MGH), Washington University, and the University of Minnesota.

ID 9 was cut in the mid-sagittal plane for other research purposes. Anyway, both hemispheres were scanned together. In this case, any fibre going to the contralateral hemisphere was considering false.

### Data acquisition

#### Dolphins

Brain scans were acquired using a 3 T MRI human whole body system (Achieva 3 T X, Philips) and a 32-channel head coil. A 3D fast acquisition with Fast Field Echo Imaging (FFE) was used to obtain high-resolution T2-weighted structural images. The parameters were: TFE factor = 105; field of view (FOV) = 150.0 × 200.0 × 150.0 mm^3^; repetition time/echo time (TR/TE) = 8.2/3.8 ms; matrix size (MTX) = 152 × 201; 150 slices with a voxel size of 1.0 × 1.0 × 1.0 mm^3^. Acquisition time per brain was 6.26 min. DW images were obtained using an Echo Planar Imaging (EPI) series with following parameters: EPI factor of 41; FOV = 224 × 168 × 150 mm^3^; TR/TE = 23,200/88 ms; MTX = 112 × 82; 75 slices with a voxel size of 2.0 × 2.0 × 2.0 mm^3^. 60 gradient directions with b = 3500 s/mm^2^ and 1 non-diffusion-weighted image (b = 0 s/mm^2^) were acquired. Acquisition time per brain was 24 min and 21 s. An additional DW image with 2 gradient direction (b = 3500 s/mm^2^) and 2 non-diffusion-weighted images were acquired in the opposite phase encoding direction for the subsequent motion/distortion correction. The protocol used at the beginning was specific for DWI image acquisition in live humans. Accordingly, various sequences were tested at the beginning by changing the b values and resolution to obtain the best SNR under those conditions. Considering all the three dolphins, the SNR, calculated as the mean signal of each gradient in the raw data, ranged for the b = 0 s/mm^2^ from 36.6 to 40 and for the b = 3500 s/mm^2^ from 13 to 16.

#### Humans

Human scans were acquired using a customised Siemens 3 T Connectom scanner, which is a modified 3 T Skyra system (MAGNETOM Skyra Siemens Healthcare) with 64-channel tight-fitting brain array coil. A 3D MPRAGE sequence was used to obtain T1w structural images with the following parameters: FOV 256 × 256 mm; TR/TE 2530/1.15 ms; voxel size of 1.0 × 1.0 × 1.0 mm^3^. Acquisition time per brain was 6.02 min. Additional T2w structural images were acquired with 3D T2-SPACE sequence with the following parameters: FOV 224 × 224 mm; TR/TE 3200/561 ms; voxel size of 0.7 × 0.7 × 0.7 mm^3^. Acquisition time per brain was 6.48 min. DW images were obtained using a Spin-echo EPI sequence with: FOV = 210 × 210 mm; TR/TE = 8800/57 ms; MTX = 140 × 140; 96 slices with a voxel size of 1.5 × 1.5 × 1.5 mm^3^. 64 gradient directions with b = 1000 s/mm^2^, 64 gradient directions with b = 3000 s/mm^2^, 128 gradient directions with b = 5000 s/mm^2^ and 2 sets of 128 gradient directions with b = 10,000 s/mm^2^ were acquired. Every 14 volumes, a b = 0 image was collected, and 1 non-diffusion-weighted image (b = 0 s/mm2) was acquired. Acquisition time per brain was 89 min.

More information on the human brain scans can be found at the following link: https://www.humanconnectome.org/study/hcp-young-adult/document/mgh-adult-diffusion-data-acquisition-details/.

### Data processing

#### Dolphins

Data were processed through FSL (https://fsl.fmrib.ox.ac.uk/fsl/fslwiki; Smith et al. 2004; Woolrich et al. 2009; Jenkinson et al. 2012) and MRtirx3 (https://www.mrtrix.org/; Tournier et al. 2012) toolboxes. Briefly, images were denoised (Veraart et al. 2015, 2016), corrected from Gibb’s ringing artefacts (Kellner et al. 2015), corrected for EPI-distortion (Holland et al. 2010), b0-field inhomogeneity (Andersson et al. 2003; Smith et al. 2004), Eddy-current and movement (Andersson and Sotiropoulos 2016). Subsequently, the dhollander algorithm was applied to compute different response functions for the white matter (WM, anisotropic), cerebrospinal fluid and grey matter (CSF and GM, both isotropic). Finally, the fibre orientation distribution (FOD) was calculated before the elaboration of the tractography (Dhollander et al. 2016a, b, 2017; Tournier et al. 2019). Each brain was then investigated individually.

#### Humans

Preprocessed data of human brains were already present in the HCP files, but further steps were added in order to create a template atlas, averaging all five subjects. Images were corrected form bias field and group DWI intensity normalisation was performed. In these data, there were 4 shells, therefore, the dhollander algorithm was used to estimate different response functions and then average them to calculate the FOD. Once all subjects’ FOD were complete, we created a population template, a template mask and warping all the FOD images to the template space (for reference, see Tournier et al. 2019).

### Tractography

**Fig. 2:**
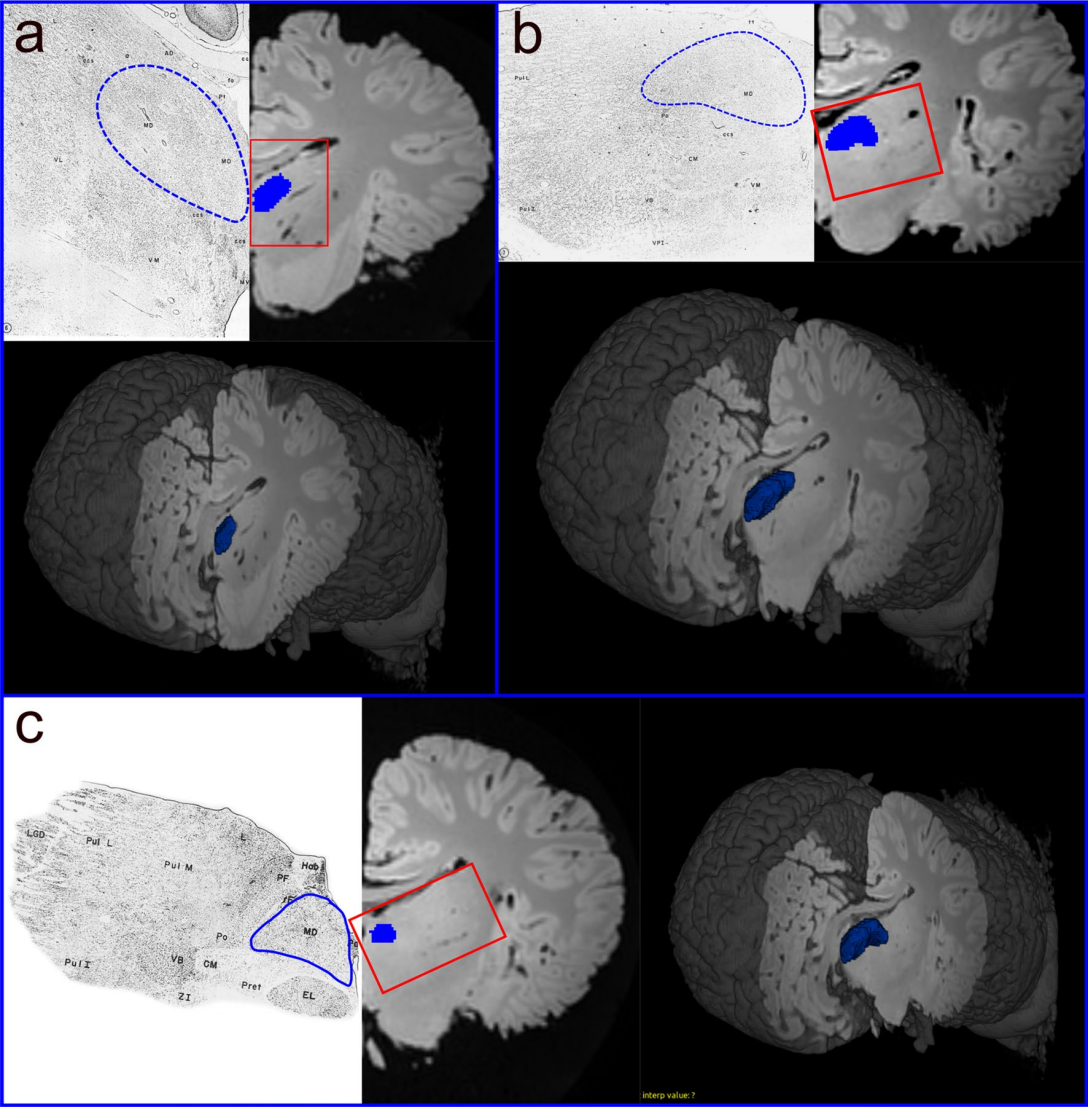
Representation of the MDN mask based on the panels descripted by Kruger (1959) and Morgane and Jacobs (1972). The directions from **a** to **c** are rostro-caudal with a 3D visualisation of the section and the whole mask at the correspondent plane; **a** section taken based on panel number 6; **b** section taken based on panel number 7; **c** section taken based on panel number 3

Throughout the following, the procedure was applied to both right and left hemispheres. In dolphins, the MDN mask was manually drawn using the ITK-SNAP software (Yushkevich et al. 2016; www.itksnap.org; version 3.8.0) following the topographical location described by Kruger (1959) and Morgane and Jacobs (1972). The thalamic subdivision is quite evident in the histological data if compared to our MRI data. We first identified the thalamic topographic position based on the histology, and then drew the areas. Since the slides did not include all the thickness of the thalamus, we tried to reconstruct the missing space following its shape (Fig. 2). The final mask was intentionally slightly eroded to avoid the generation of erroneous extra fibres. Since there is no map of the PFC location in *Cetacea*, a PFC area was first created, based from the tracts originating from the MDN. To verify that the tracts were not artefactual, we restricted the tracking using the designated PFC as the seed image and reaching the MDN nucleus, thus restraining the fibres to the bundle only connecting one with the other.

In the human, MDN and PFC were manually drawn with ITK-SNAP, based on the currently available templates and atlases (Tamraz and Comair 2000; Cho 2010; Ding et al. 2016). For both dolphin and human brains, fibre tracking was performed through a deterministic algorithm based on CSD, with the FOD file as input and the following parameters: FA threshold 0.1 for dolphin brains (as they were fixed) and 0.2 for human brains (since the subjects were alive), step size 0.1 mm, default angle threshold 60°, and default streamline count (5000).

## Results

### Seeding point: MDN nuclei

**Fig. 3:**
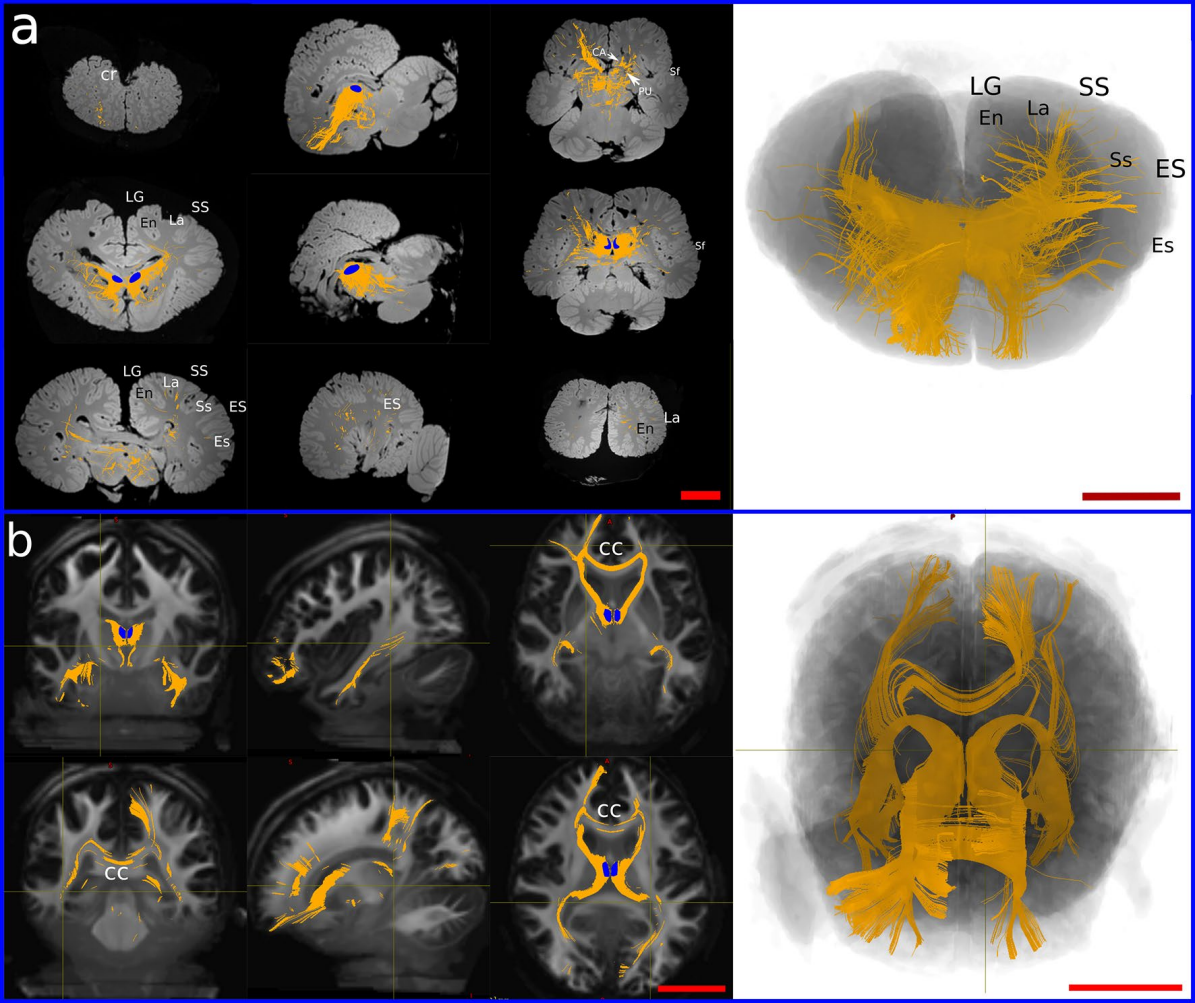
Prefrontal pathways in the dolphin and human brains. **a** Tracts generated from seeding the MDN (blue shape) in dolphin. **b** Tracts generated from seeding MDN (blue shape) in human. *CC* corpus callosum, *En* entolateral sulcus, *Es* ectolateral sulcus, *ES* ectolateral gyrus, *La* lateral sulcus, *LG* lateral gyrus, *Sf* sylvian fissure, *Ss* suprasylvian sulcus, *SS* suprasylvian gyrus. Red scale bar = 5 cm

In the dolphin brains, the main fibre bundles exiting the MDN were directed cranially running below the cruciate sulcus (Cr) towards the ventro-cranial pole, passing between the Putamen (PU) and caudate nucleus (CA). Other consistent fibre bundles went dorsally following the internal capsule and terminated in i) the supralimbic cortex around the entolateral sulcus (En), the lateral sulcus (La), and the suprasylvian sulcus (Ss); then ii) laterally in the temporal lobes around the ectolateral sulcus (Es). Some tracts ran caudally to the Edinger–Westphal nucleus (EW), the Interstitial Nucleus of Cajal (INC) and the elliptic nucleus (NE), then moved ventrally towards the crus cerebri (Fig. 2a). Projections from the MDN were also commonly distributed within the thalamus and then ran to other brain areas. Few aberrant fibres passed through the superior colliculi (SC) (Fig. 3a).

In the human brain, fibres ran to the PFC following the anterior thalamic peduncle, then went laterally to the temporal lobe following the inferior thalamic peduncle. Some of the latter fibres split from the inferior thalamic bundle to join the optic radiation directed towards the parietal lobe. We also noted fibres that reached the CC to spread in the contralateral hemisphere (Fig. 3b).

**Fig. 4:**
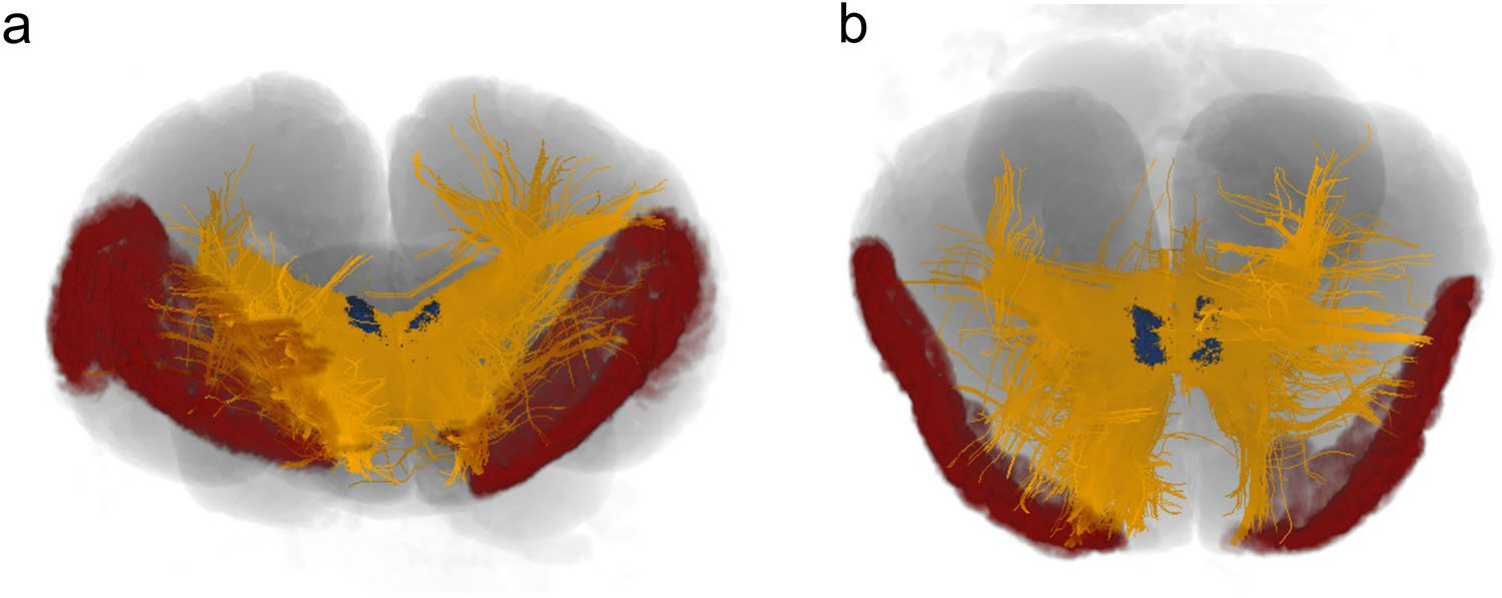
Putative PFC (red mask) based on previous projections from the MD (blue mask). **a** Frontal view; **b** dorsal view

Thanks to our previous results, we were able to estimate a presumptive frontal region based on the extension of the fibres on the WM tracts (Fig. 4).

### Seeding point: acquired PFC

**Fig. 5:**
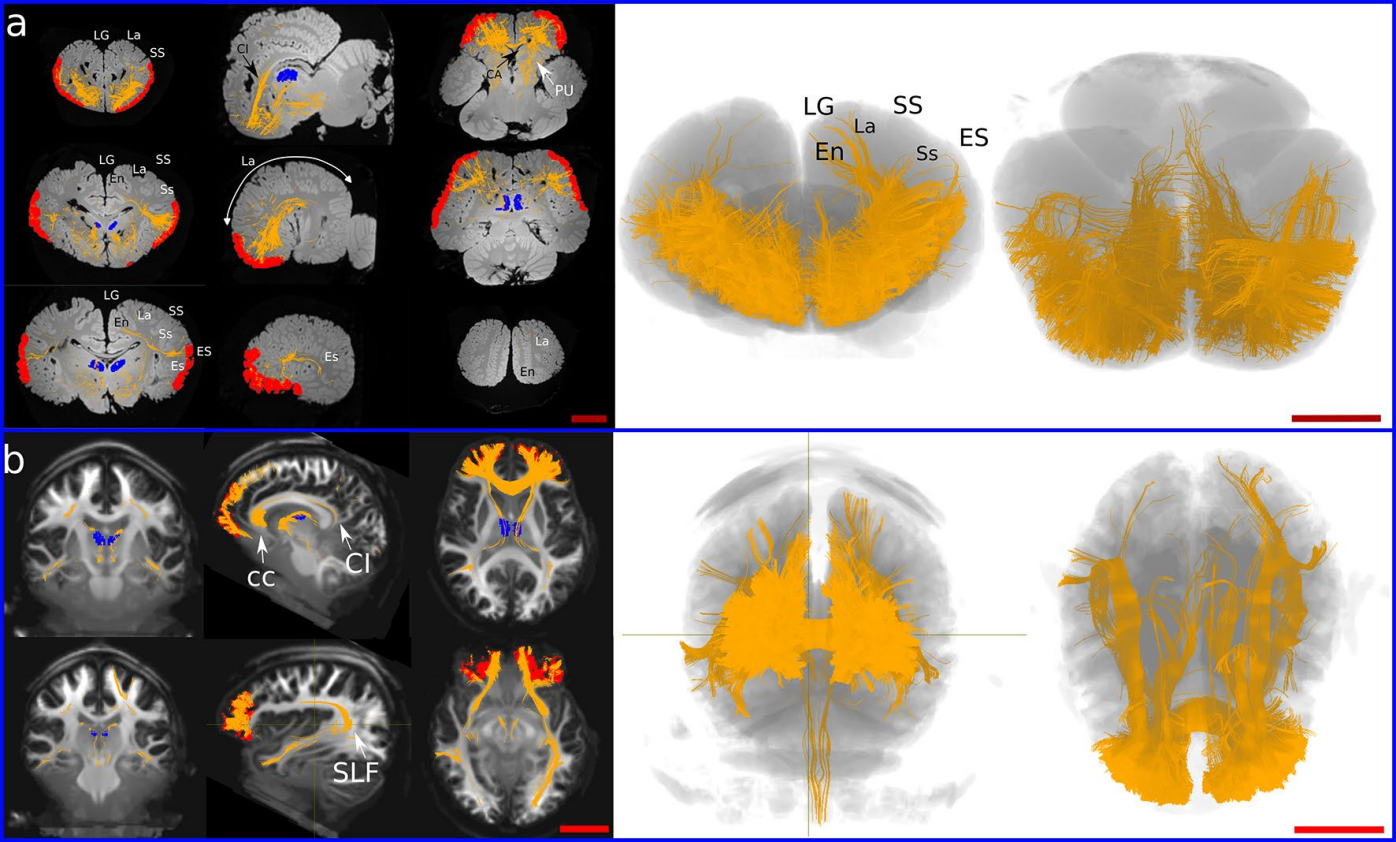
Prefrontal pathways in the dolphin and human brains. **a** Tracts generated from seeding the PFC (red shape) in dolphin. **b** Tracts generated from seeding PFC (red shape) in human. The MDN is represented in blue. *CA* caudate nucleus, *CC* corpus callosum, *CI* cingulum, *En* entolateral sulcus, *Es* ectolateral sulcus, *ES* ectolateral gyrus, *La* lateral sulcus, *LG* lateral gyrus, *PU* putamen, *SLF* superior longitudinal fasciculus, *Ss* suprasylvian sulcus, *SS* suprasylvian gyrus. Red scale bar = 5 cm

Seeding the putative PFC resulted in the presence of several ipsilateral U-shaped fibres within the area. Other fibre tracts were oriented towards the basal ganglia and the cingulate cortex (CI). Other bundles continued through the MDN to end in the pons. A consistent number of fibres joined the superior longitudinal fasciculus (SLF) directed to the temporal lobe (Es, Ss) while others arose to En and La (Fig. 5a). Finally, some tracts reached the CC and crossed contralaterally.

In the human brain, ipsilateral fibres created from the PFC joined the inferior fronto-occipital fasciculus and reached the V1. Other fibres joined the fornix, then ran caudally towards the MDN, the CI. Some tracts were directed to the temporal lobe apparently through the SLF. Fibres directed towards the mesencephalon reached the periaqueductal grey and the red nucleus in the tegmentum (Fig. 5b). Finally, consistent fibre bundles moved to the CC and then crossed to the contralateral hemisphere.

### Specifically constrained thalamo-cortical connections

To delimit the fibres previously generated singularly from the MDN nuclei or from the presumptive PFC, we also selectively constrained the tracking between these two regions.

**Fig. 6:**
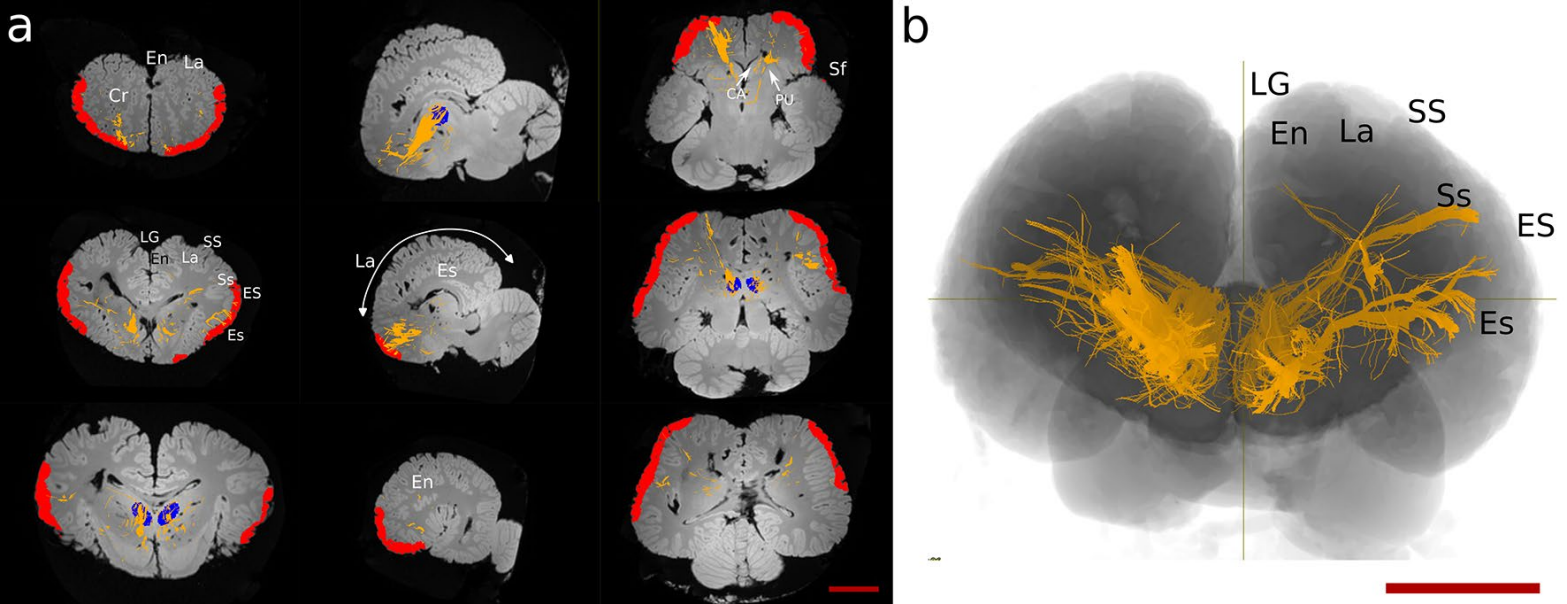
Constrained tractography between the putative found PFC (red shape) and the MDN (blue shape). *CA* caudate nucleus, *Cr* cruciate sulcus, *En* entolateral sulcus, *Es* ectolateral sulcus, *ES* ectolateral gyrus, *La* lateral sulcus, *LG* lateral gyrus, *PU* putamen, *Sf* sylvian fissure, *Ss* suprasylvian sulcus, *SS* suprasylvian gyrus

In general, these fibres were fewer and more limited. In the dolphin, cortically seeded bundles passed below the Cr and between the basal ganglia to reach the MDN. Other streamlines ran laterally to the temporal lobe (Ss, Es), and very few fibres continued dorsally to the ENs and LAs. Finally, some fibre bundles were directed caudally to the red nucleus and the elliptic nucleus (Fig. 6).

**Fig. 7:**
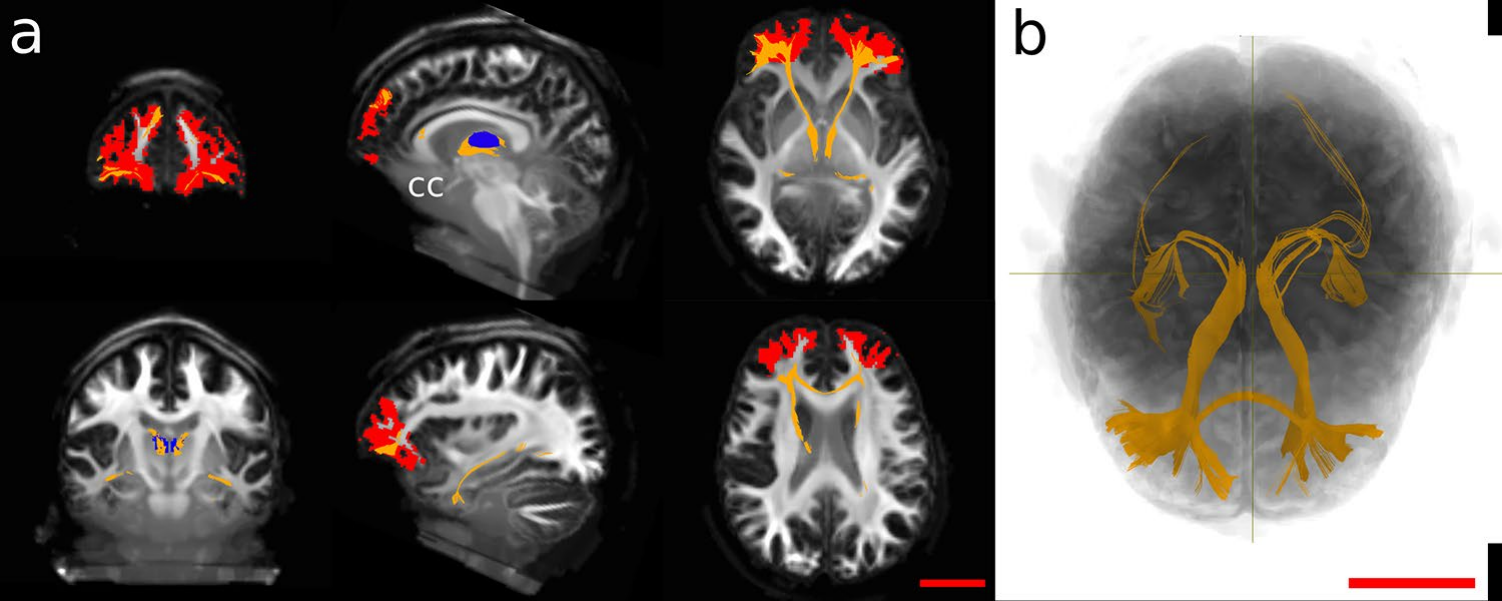
Constrained tractography between the putative found PFC (red shape) and the MDN (blue shape). *CC* corpus callosum

Prefrontal pathways in the human brain between PFC and MDN also crossed through the CC and reached the temporal lobe of the contralateral hemisphere through the inferior thalamic peduncle. Few fibres detached from the inferior thalamic peduncle and continued caudally until the VC (Fig. 7).

## Discussion

A precise identification of the topography and connectivity of the PFC (or its homologue) in dolphins represents a consistent step forward towards the understanding of their brain architecture and the neural basis for some of the complex behaviours of the species. To the best of our knowledge, DWI technique is currently one of the very few technically feasible and ethically acceptable approaches to identify the PFC in dolphins, and potentially other large ex-vivo brains.

In the present study, we performed CSD-based tractography and aimed at identifying fibre tracts that connect selected thalamic nucleus to their related cortical targets/origin. We then compared the data with those obtained in the human brain. Since the human PFC is one of the key areas assigned to higher brain functions (Tranel et al. 2003; Butler and Hodos 2005; Kandel et al. 2021), we searched for an area in the dolphin brain with the same characteristic connections.

CSD is a HARDI algorithm of DWI that, within the limits of human clinical MRI parameters, can reconstruct crossing fibres within a voxel, thus giving a more plausible biological result (Arrigo et al. 2016; Jeurissen et al. 2017). The algorithm can be applied and extended also to fixed brains, much like with DTI (D’Arceuil and de Crespigny 2007; Rane and Duong 2011; Gerussi et al. 2022). Classical retrograde and anterograde tract-tracing remains the gold standard methodology to accurately study brain connections. However, the technique was seldom performed in cetacean brains, and likely will not be in the future because of the ethical constraints briefly outlined in the Introduction. In this context, opportunistic fixed-brain DWI (and its various algorithmic variations), therefore, constitutes an adequate method to investigate brain connections in these mammals, even considering its biases and limits to interpretation (Jeurissen et al. 2017; Schilling et al. 2020). Fixation produces microstructural changes such as dehydration or tissue degeneration, which in turn may alter some MRI parameters including SNR, FA and apparent diffusion coefficient (ADC) (D’Arceuil and de Crespigny 2007; Rane and Duong 2011). Therefore, the obtained results must consider all the limitations of DWI in general and DWI applied to fixed tissues.

### Tracing and tractography in dolphins and porpoises

Literature on evoked potential placed the bottlenose dolphin V1/V2 around En in the LG, extending rostro-caudally and with the La as its lateral boundary. A1/A2 develops from there along the SS, reaching the Ss with a rostro-caudal direction (for review see Supin et al. 2001). According to the literature, the Cr separates rostrally the medial SSC from the lateral MC. Other reports that used tracing in the harbour porpoise (*Phocoena phocoena*) established the existence of projections from the parvocellular part of the MGN to the suprasylvian and ectosylvian gyri (Krashnoshchekova and Figurina 1980; Voronov et al. 1985), and the involvement of the LGN as target of the optic nerve. Evoked potentials were used also for track-tracing-based injections in various areas of the neocortex (LG, the SS, ES, temporal and orbital) of the harbour porpoise (Revishchin and Garey 1990). Overall, the data obtained showed that medial thalamic projections progressively crossed to a lateral cortical position, therefore, ending contralaterally to their origin (see Revishchin and Garey 1990, Fig. 13). However, no tracing study was ever performed on a putative PFC.

A recent DTI study described for the first time the existence of a direct auditory pathway from IC to MGN to the temporal lobe near the Sylvian fissure in the common (*Delphinus delphis*) and pantropical spotted dolphin (*Stenella attenuata*), and an overlap of thalamic visual and auditory pathways (Berns et al. 2015). Such direct pathways, often hypothesised, but never demonstrated before, suggest a direct and profound interconnection of the dolphin visual and auditory system. Nevertheless, given the extremely limited data concerning the functional auditory region in cetaceans, it should be considered a still partially unsolved scientific question.

### Topography and characterisation of the PFC in the human and dolphin brain

**Fig. 8:**
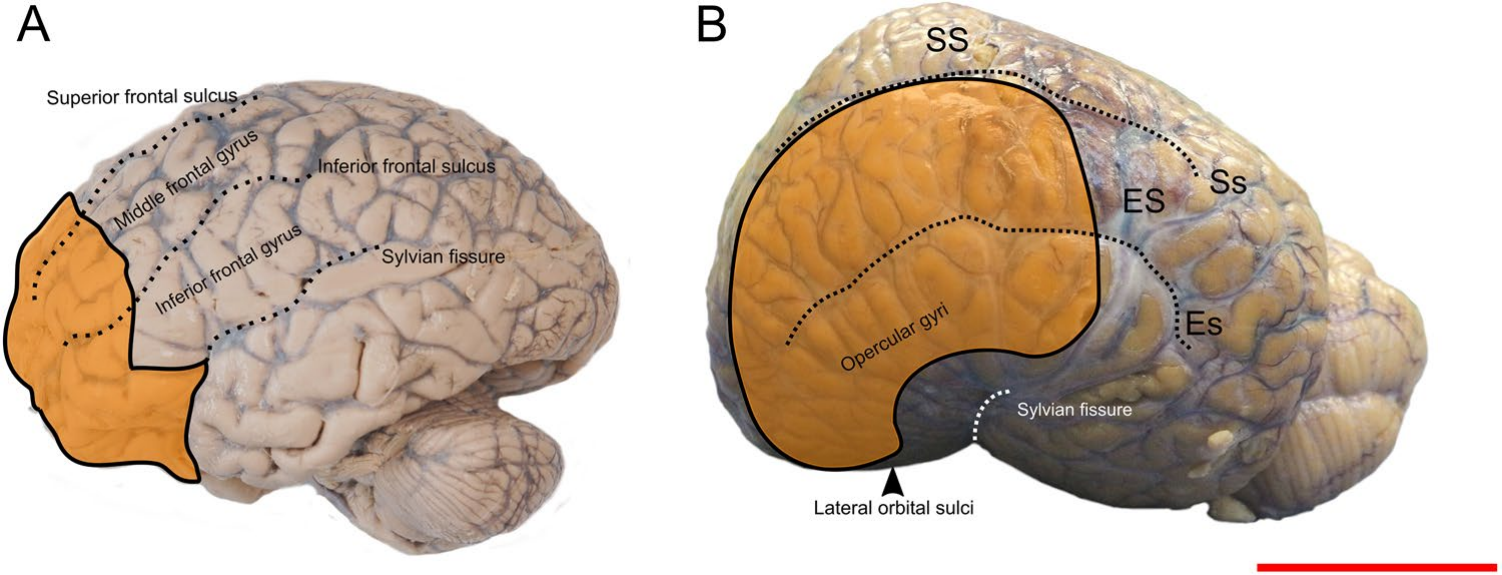
Approximate PFC representation (orange area) in human (**A**) and dolphin (**B**) brain. For abbreviations, see the list. Red scale bar = 5 cm

The human PFC can be divided into four functional areas: orbitofrontal cortex (OFC), dorsolateral PFC (dlPFC), dorsomedial PFC (dmPFC) and ventromedial PFC (vmPFC) (Kolb 2015). The caudolateral boundaries of the PFC roughly correspond to the cranial part of the precentral sulcus (premotor cortex) and medially to the cingulate cortex. The Brodmann areas overlapping the human PFC are: BA8-14, 24, 25, 32, 44–47 (Murray et al. 2017). These are marked by notable cytoarchitectural differences that determine their boundaries (Brodmann’s 2006; Petrides and Pandya 2012). Although the frontal lobe possess its own cytoarchitectural peculiarity (Hof et al.  2005), there are no such characteristic differences in the bottlenose dolphin, and other cetaceans in general (Kern et al. 2011; van Kann et al. 2017). We started by identifying topographical landmarks that may characterise comparatively the dolphin PFC (see orange area in the Fig. 8) and then proceeded with CSD tractography. The average mass of the brain in the bottlenose dolphin is 1.550 g (Huggenberger et al. 2019), vs. the average value of 1300–1400 g for the human (Miller and Corsellis 1977) must be taken in account. Without discussing the body and brain weight correlation, or the Encephalization Quotient^1^ (Jerison 1973; for discussion of its application to cetaceans see Cozzi et al. 2016), we emphasise that the position of the area occupied by the PFC, based on topographically equivalent landmarks, appears more lateral in the dolphin brain comparatively to man, even considering the different brain shape and dimensions.

### Prefrontal pathways

In the human DWI scans, seeding the human MDN (Fig. 2b) resulted in fibres that reached most of the PFC through the anterior thalamic peduncle, while some other fibres joined the inferior thalamic peduncle and were then oriented towards the somatosensory association area in the parietal lobe and V1. Our results were consistent with those found by Grodd et al. (2020). When the same method was applied to the brains of the bottlenose dolphins, the resulting fibres joined the internal capsule, passed between the basal ganglia towards the ventro-cranial pole and ended ventrally to the Cr. Other fibres were directed towards either the LGN or the MGN, going through the internal capsule and terminating in the parietal lobe. We also noted that some fibres were directed caudally, possibly to merge in post-thalamic tectal pathways. A putative PFC could be identified in the bottlenose brain by mapping fibres that reached the non-parietal neocortex (Fig. 8). The projections arising from this putative PFC then continued (a) within the PFC itself; (b) moved to the contralateral PFC through the CC; (c) to the CI; (d) to the pons passing between the basal ganglia and MDN; or (e) to the temporal lobe following the SLF. The connectivity pattern detected in the bottlenose brain was in fact similar to that found in the human brain.

To check whether these fibres were not just artefactual, we first seeded the MDN to establish where the fibres were going. Then we seeded the designated area to see if some of the fibres projected independently back to the MDN. We noticed that some fibres again connected the PFC with the MDN, but other bundles were oriented towards different areas. The PFC is a multimodal association area which receives and sends inputs to other brain regions, and—to this effect—our results were consistent with what reported in the literature (Fuster 2015). We finally constrained the seeding between the two areas to qualitatively see the amount of fibres only related to these seeds, and exclude other unspecific bundles. We found a more marked asymmetry in the dolphin brains compared to human brains (Figs. 6 and 7), but given the scope of our study and its qualitative approach, we did not emphasise this aspect.

The acquisition parameters used to scan the live human brains (referred to the HCP) were evidently better than those of the (dead) dolphin brains. However, we detected no substantial loss of signal in the formalin-fixed dolphin brains, so that identification of a putative PFC area in their brain gave results that were largely comparable to the human. No former tracking or injection studies based on evoked potential reported a functional identification of a putative PFC in dolphin, hence, this is the first report of a putative PFC in this species, and, to the best of our knowledge, in all cetaceans. Its boundaries start in the orbital lobe, extend laterally in the cranial ES and reach the cranial opercular lobe. However, additional fibres were connected to areas placed below the SSC and MC cortices, in which pyramidal neurons were previously found (Manger 2006).

According to the concept of the “initial brain” (for reference see Glezer et al. 1988, Fig. 7) modern mammals still retain some key topographical features already present in the brains of their early ancestors, notwithstanding the divergent evolutionary path of the taxa. The enlarged human neocortex contains far more and varied cortical modules than in primordial mammals. The cetacean brain obviously followed a different evolutionary route since the brain mantle greatly expanded to the uttermost known limits for mammals in terms of relative cortical size, while the cytoarchitecture is poorly differentiated and mostly homogeneous in the neocortex (Cozzi et al. 2017).

**Fig. 9:**
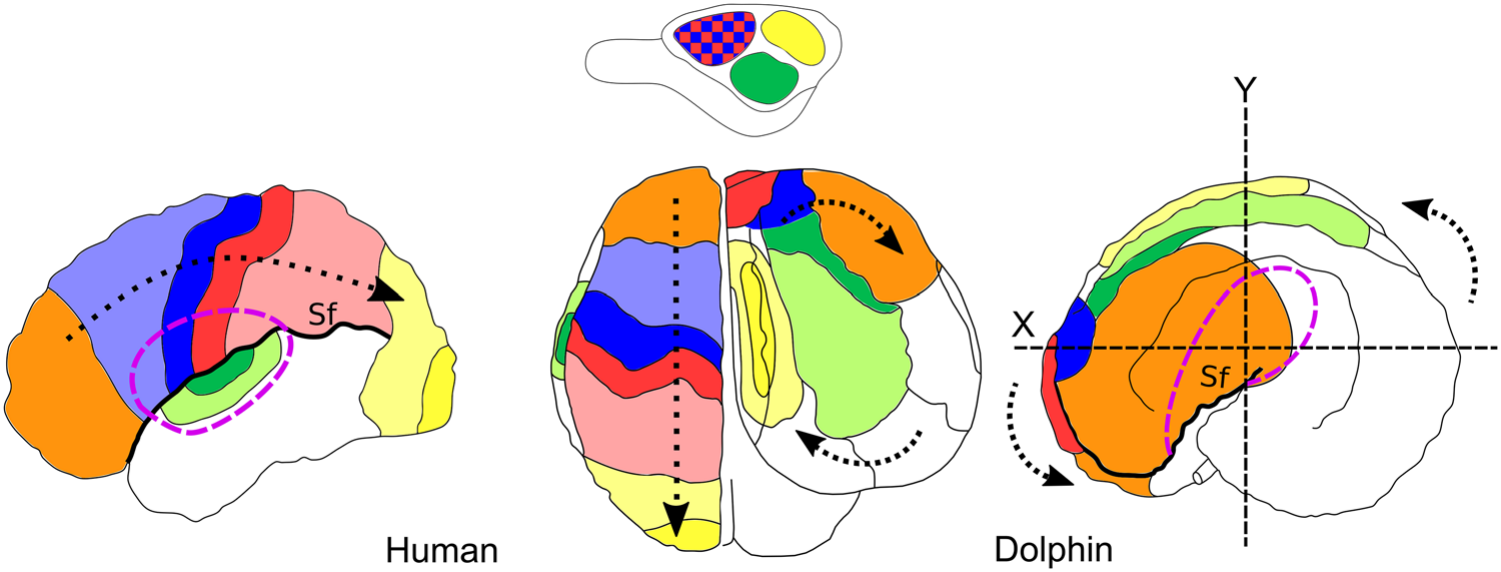
Schematic picture of the brain evolution from its “initial” form (top, Glezer et al. 1988) to the dolphin (Jacobs et al. 1984; Cozzi et al. 2017) and human (Martin 2021) brains (bottom). Orange, PFC; Blue, SSC; Red, MC; Green, A1/A2; Yellow, V1/V2. Sf, Sylvian fissure. Dashed purple line delimits the border of the insular cortex. The dotted arrow in the human brain indicates the displacement of the other areas due to the expansion of the PFC. The dotted arrows in the dolphin brain indicate the rotation of the organ around the insular cortex and the probable cranio-lateral shift of the PFC and other cortical areas on the surface. X, Y body axes

In the human brain, the development of the PFC displaced all the areas caudally, thus modifying the primeval topographical scheme (Fig. 9). In cetaceans, the progressive evolution to life in the water caused a wide range of body structure modifications. In the head specifically, the growth of the melon, the telescoping process, and the nasal shift which caused the change of the cranial axes and shape, might have prevailed over the longitudinal development of the brain (Miller 1923; Cozzi et al. 2017; Roston and Roth 2019). Consequently, the brain folded around the insula (see dotted arrows in Fig. 9) and expanded more laterally: what in terrestrial mammals is “caudal” becomes “dorsal” in the dolphin and so forth (Morgane and Jacobs 1972). Concomitant space limits within the cerebral cavity prompted a striking cortical gyrification and induced the topographical shift of neocortical areas. Our data based on CSD tractography confirmed that the (evolutionary) process that modified the cetacean brain was compatible with the persistence of a very large and richly connected PFC area.

The PFC of terrestrial mammals, and the correspondent areas in birds, are highly innervated by dopaminergic neurons (Sawaguchi and Goldman-Rakic 1991; Gaspar et al. 1992; Güntürkün 2005). Based on this neurochemical concept, further studies are needed to investigate the actual presence of dopaminergic neurons in the PFC of dolphins. This is particularly important in the fronto-ventral area, where one should not expect to find an associative cortex (Manger 2006, referring to Kojima 1951), and in the lateral (opercular) area. Additional functional MRI (fMRI) or recently developed functional near-infrared spectroscopy (fNIRS) techniques (Scholkmann et al. 2014) might also become useful for the study of brain functions through the proxy of increased blood flow during local stimulation, very similar to blood-oxygen-level-dependent contrast in fMRI. A composite multidisciplinary approach may eventually shed light on the unmapped cortical fields of these mammals with remarkable cognitive abilities.

## Data Availability

The datasets generated during and/or analysed during the current study are available from the corresponding author on reasonable request.

## Abbreviations

En: Entolateral sulcus
Es: Ectolateral sulcus
La: Lateral sulcus
Sf: Sylvian fissure
Ss: Suprasylvian sulcus
AC: Anterior commissure
CC: Corpus callosum
Cl: Cingulate cortex
CSD: Constrained spherical deconvolution
DTI: Diffusion tensor imaging
DWI: Diffusion-weighted imaging
ES: Ectolateral gyrus
MC: Motor cortex
MDN: Medio-dorsal thalamic nucleus
MGN: Medial geniculate nucleus
LG: Lateral gyrus
LGN: Lateral geniculate nucleus
OT: Optic tract
PFC: Prefrontal cortex
SS: Suprasylvian gyrus
SSC: Somatosensory cortex

## Footnotes

1 EQ [EQ = E^i^ / 0.12 × P^2/3^; E.^i^ = mean brain weight, P = mean body weight] is 3.79 in the bottlenose dolphin *vs*. 6.62 in man, according to Huggenberger et al. (2019)
